# Aligning Open‐Access Surgical Education With Need: Curriculum Coverage and Gaps in the United Nations Global Surgery Learning Hub (SURGhub)

**DOI:** 10.1002/wjs.70533

**Published:** 2026-08-13

**Authors:** Dáire Goodman, Sara Mohan, Pauline Wake, Dolorence Wakida, Dara Kavanagh, Juan Carlos Puyana, Eric O'Flynn

**Affiliations:** ^1^ Institute of Global Surgery School of Population Health Royal College of Surgeons in Ireland Dublin Ireland; ^2^ UCD Perinatal Research Centre University College Dublin National Maternity Hospital Dublin Ireland; ^3^ School of Medicine and Health Sciences Boroko National Capital District Papua New Guinea; ^4^ School of Nursing Uganda Martyrs University Nkozi Uganda; ^5^ Department of Surgical Affairs Royal College of Surgeons in Ireland Dublin Ireland

**Keywords:** E‐learning, education, global surgery, open‐access, training

## Abstract

**Background:**

The United Nations Global Surgery Learning Hub (SURGhub) is a community‐led, open‐access digital platform providing quality‐assured surgical education, particularly in low‐resource settings. However, growth in course numbers and learner reach does not necessarily ensure balanced coverage of competencies required in early training. We mapped SURGhub content against selected early training curricula to identify priority gaps and establish a baseline for educational improvement.

**Methods:**

We conducted a cross‐sectional curriculum mapping study of SURGhub courses available in January 2026. Courses classified as Level 1 or 2 were mapped against selected benchmark curricula in surgery, anesthesia, obstetrics and gynecology (OBGYN), and perioperative nursing. Reviewers applied a three‐component rubric assessing relevant content quantity, coverage breadth, and early training essentiality. Component scores were summed to produce a total score from 0 to 6, with domains classified as critical, high‐, moderate‐, or low‐priority curriculum gaps.

**Results:**

A total of 82 eligible courses were evaluated across 99 curriculum domains. Mean domain scores were highest for surgery at (3.67/6), followed by anesthesia (2.53), OBGYN (0.84), and perioperative nursing (0.83). Coverage was broad but uneven. Practical perioperative topics, trauma, patient safety, and procedural skills were better represented than foundational sciences and several specialty‐specific clinical domains. Critical gaps were identified in one of 12 surgical domains, five of 19 anesthesia domains, eight of 12 perioperative nursing domains, and 37 of 56 OBGYN domains.

**Conclusions:**

SURGhub demonstrates the potential of open‐access, low‐bandwidth digital education to expand access to surgical learning globally, but Levels 1 and 2 course coverage was uneven across selected benchmark curricula. Approximately two‐thirds of evaluated OBGYN and perioperative nursing domains were classified as critical gaps. This mapping establishes a baseline for curriculum‐aligned content commissioning and repeated evaluation. Future research should evaluate course uptake, completion, and learner outcomes following targeted content development while maintaining attention to accessibility, engagement, and equity.

## Graphical abstract

The United Nations Global Surgery Learning Hub (SURGhub) is a community‐led, open‐access digital platform providing quality‐assured surgical education, particularly in low‐resource settings. However, growth in course numbers and learner reach does not necessarily ensure a balanced coverage of competencies required in early training. We mapped SURGhub content against selected early training curricula to identify priority gaps and establish a baseline for educational improvement.

## 1 Introduction

Since the 2015 Lancet Commission on Global Surgery, surgical, obstetric, and anesthesia care have increasingly been recognized as essential components of universal health coverage. However, an estimated 160 million necessary surgical procedures continue to go unperformed each year, and the mortality associated with surgery is estimated at 3.5 million adult deaths, whereas 50 million people experience postoperative complications globally each year [1]. This unmet need is concentrated in low‐ and middle‐income countries (LMICs), where case‐fatality rates for otherwise treatable conditions such as obstructed labor, trauma, and common abdominal emergencies remain unacceptably high and contribute substantially to avoidable death and disability [2]. A key challenge is not just the insufficient number of trained providers in LMICs but also the lack of educational resources available for their training and professional development. Access to online educational content is hampered by absent or unstable internet connectivity, regional inequity of access to resources, inappropriate resources for their context and training, and a lack of independent quality‐controlled standardized content [3].

The United Nations Global Surgery Learning Hub (SURGhub) platform [4] was launched in June 2023 through a partnership between the United Nations Institute for Training and Research (UNITAR), the Global Surgery Foundation, and the Royal College of Surgeons in Ireland, in association with the Johnson & Johnson Foundation. SURGhub is a community‐led, open‐access digital education platform designed for the global surgical team with low‐bandwidth functionality, mobile access, and offline capability intended to reduce barriers to learning in resource‐constrained settings. Its courses are quality‐assured through multidisciplinary expert review and span surgery, anesthesia, obstetrics and gynecology (OBGYN), and perioperative nursing [5]. As of March 2026, the platform hosted 135 courses with over 40,000 learners from 195 countries enrolled. SURGhub primarily curates pre‐existing courses contributed by international academic, professional, and nongovernmental organizations. Potential courses are identified by the SURGhub project team or submitted by organizations. The project scope, content, and technical committees assess the proposed content and are further supported by dedicated content and technical review panels.

Each course is reviewed by at least four experts, two of whom are specialists in the relevant topic, whereas content reviewers are also required to have experience practicing in a resource‐constrained setting (RCS) [3]. Level 1 content is intended for nonspecialists, Level 2 for early specialty trainees, and Levels 3 and 4 for specialists and subspecialists, respectively.

Although early evaluations of SURGhub reported broad uptake and learner satisfaction [3], the extent to which current SURGhub content systematically covers the core competencies expected in recognized training programs for the surgical team has not formally been assessed. An increase in the number of courses and learners does not necessarily demonstrate that the available content provides a balanced coverage of the competencies expected of a trainee during their early stage careers. As SURGhub was developed as a platform incorporating educational content from multiple global contributors rather than a comprehensive curriculum developed for the surgical team, its portfolio may contain under‐represented foundational or discipline‐specific domains.

Curriculum mapping systematically compares educational content against defined curricular expectations to identify curriculum gaps and guide future course development. This approach has been used to identify curriculum gaps and guide course prioritization in educational systems [6]. Curriculum mapping has also been described as a mechanism to identify curriculum gaps across medical school curricula and providing a practical roadmap for future course prioritization and commissioning [7].

The aim of this cross‐sectional curriculum mapping study was therefore to map SURGhub Level 1 and Level 2 courses against selected international benchmark training curricula in surgery, anesthesia, OBGYN, and perioperative nursing, identify critical and high‐priority curriculum gaps, and establish a baseline to inform future educational improvement and platform expansion.

## 2 Materials and Methods

We conducted a cross‐sectional curriculum mapping study of SURGhub courses available in January 2026 to assess alignment with selected benchmark curricula and establish a baseline for educational improvement. Reporting was informed by applicable Standards for QUality Improvement Reporting Excellence in Education (SQUIRE‐EDU) items [8].

One subject‐specific reviewer mapped each discipline. The characteristics of the four reviewers are as follows: a Plastic Surgery Specialist Registrar in Ireland with 8 years clinical and teaching experience and currently a doctoral candidate investigating the SURGhub platform, an OBGYN Research Fellow and Specialist Registrar in Ireland with 10 years of clinical and teaching experience, an anesthesiologist and clinical lecturer in Papua New Guinea with 20 years of clinical and teaching experience, and a Uganda nurse educator and Head of a Public Health Nurses College and clinical lecturer practicing in Uganda with 23 years of clinical and teaching. The latter three are SURGhub project scope committee members. Reviewer profiles are provided in Table S1.

Our evaluation focused on courses classified as Levels 1 and 2 as these levels are aimed at foundational and early specialty education and therefore aligned with the stages of training represented by the selected benchmark curricula. Level 3 and Level 4 courses were excluded because they would require advanced and subspecialty‐specific curriculum benchmarks. The 82 courses and their discipline mappings are provided in Table S2.

Curricula were purposively selected as structured, domain‐based frameworks with competencies sufficiently detailed for course‐level mapping and relevant to SURGhub's geographical and practice contexts.

The UK and Ireland Intercollegiate Membership of the Royal College of Surgeons (MRCS) curriculum and Royal College of Physicians of Ireland (RCPI) Institute of Obstetrician & Gynecologists Basic Specialist Training curriculum provided established frameworks for early surgical and OBGYN training. The University of Papua New Guinea (PNG) anesthesia curriculum provided an early training framework from a resource constrained setting. The International Federation of Nurse Anesthetists (IFNA) curriculum provided an internationally recognized framework for nurse anesthesia education within perioperative nursing. These were practical benchmarks rather than universal reference standards. Curriculum domains were retained but closely related sequential domains were combined where they represented a continuous topic area.

Reviewers assigned relevant courses to curriculum domains. As no validated instrument for comparing an online course portfolio with multiple discipline‐specific training curricula was identified, we developed a three‐component curriculum gap prioritization rubric assessing quantity of relevant SURGhub content, breath of curriculum‐domain coverage, and the importance of the domain to early training. The rubric was designed to provide a simple and consistently applicable method for assessing curricula coverage according to three related categories: Quantity was scored 0 for no relevant courses, 1 for some relevant courses but limited in number, or 2 for substantial number of relevant courses. Breadth of coverage was scored as 0 where major components of the domain were absent, 1 for partial coverage, or 2 for good coverage of the principal topics. Essentiality was scored 0 for essential or core, 1 for important, or 2 for helpful but not core. The third component was intentionally scored in this direction so that limited coverage of an essential domain produced a lower total score and therefore a higher curriculum‐gap priority. Composite curriculum gap scores were sued as descriptive summary measures of curriculum coverage, whereas means and medians were used to facilitate comparisons across disciplines. No inferential statistical analyses were performed.

Component scores were summed from 0 to 6. 0–1 were classified as critical gaps, 2–3 as high‐priority gaps, 4 as moderate‐priority gaps, and 5–6 as low‐priority gaps. The rubric was reviewed by the four discipline reviewers and applied to exemplar domains to establish shared interpretations. Ratings considered unclear by the assigned reviewer were discussed among the wider review team until agreement was reached. The rubric was developed as a prioritization tool rather than a validated measure of curriculum completeness or educational quality and did not undergo formal psychometric validation or inter‐rater reliability testing.

## 3 Results

A total of 82 unique Level 1 and Level 2 courses were mapped across 99 curriculum domains. Courses could be mapped to multiple disciplines and domains; therefore, discipline‐specific course counts were not mutually exclusive. Mean curriculum‐gap prioritization scores were highest for surgery at (3.67/6), followed by anesthesia (2.53), OBGYN (0.84), and perioperative nursing (0.83). Median scores were 3.5, 3.0, 0, and 0, respectively (Table 1). The proportional distribution of priority classifications is shown in Figure 1. Critical gaps accounted for 8.3% of surgical domains, 26.3% of anesthesia domains, 66.7% of perioperative nursing domains, and 66.1% of OBGYN domains.

**TABLE 1:** Summary of curriculum‐gap prioritization scores by discipline.

Discipline	Domains mapped	Mean score	Median score	Critical gaps	High gaps	Moderate gaps	Low gaps
Surgery	12	3.67	3.5	1	5	2	4
Anesthesia	19	2.53	3	5	8	5	1
Perioperative nursing	12	0.83	0	8	4	0	0
OBGYN	56	0.84	0	37	18	1	0

**FIGURE 1:**
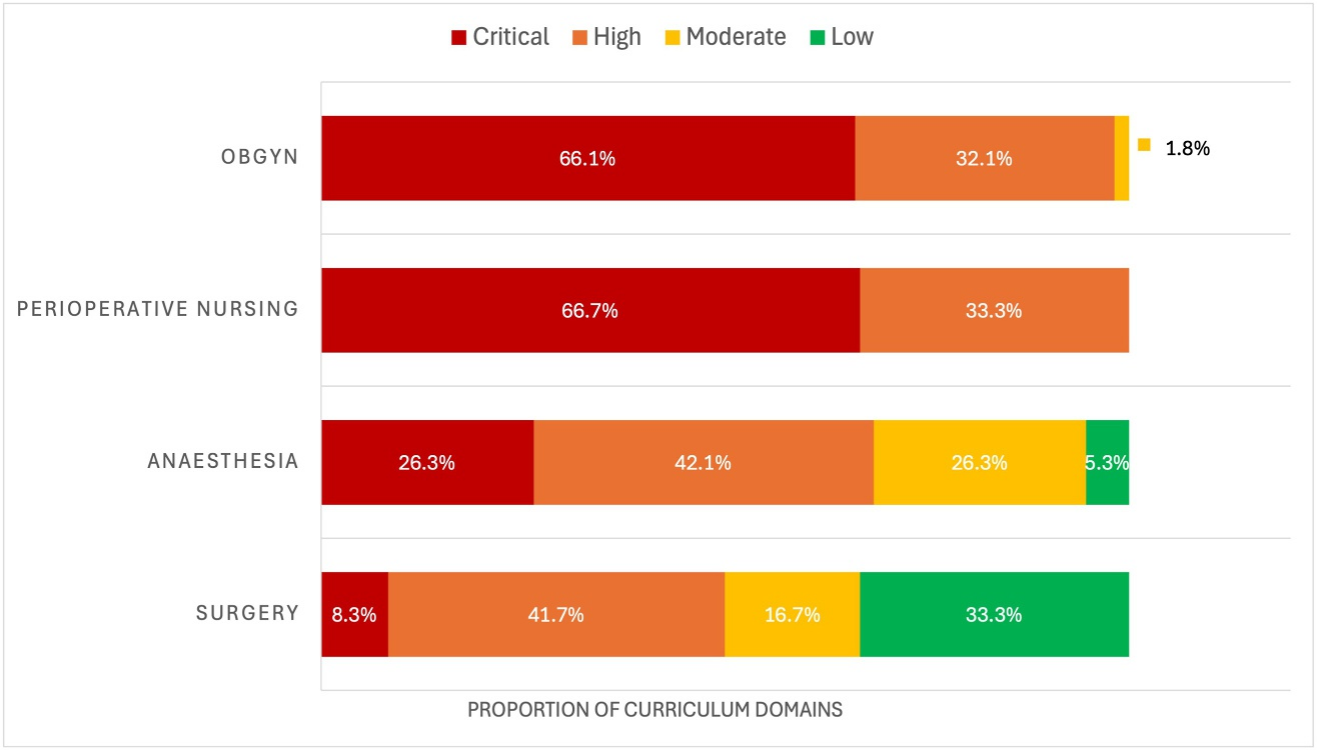
Distribution of curriculum‐gap priorities by discipline. Stacked bars show the proportion of curriculum domains classified as critical‐, high‐, moderate‐, or low‐priority gaps within each discipline. Percentages were calculated using the total number of evaluated domains within each discipline as the denominator.

### 3.1 Surgical Curriculum Coverage

Across the Intercollegiate MRCS domains, SURGhub Level 1 and Level 2 courses demonstrated uneven coverage (Table 2) with relatively greater representation of trauma, perioperative care, and technical skills compared with basic sciences and pediatric surgery. Trauma was classified as a low‐priority gap, whereas perioperative management was classified as a moderate‐priority gap and clinical and procedural skills as low‐priority gaps. In contrast, domains in applied surgical anatomy, physiology, pathology, and imaging and data/audit had 2–4 courses each and were classified as high‐priority gaps. The domain focusing on surgical care of children was designated as having a critical‐priority curriculum gap with only 3 applicable courses. Medico‐legal and patient safety, communication and professionalism, and microbiology had moderate or low gap ratings, indicating higher representation of nontechnical skills and safety content in the current portfolio.

**TABLE 2:** Level 1 and Level 2 surgery course mappings to MRCS curriculum domains.

Domains	Count of applicable courses	Relevant content quantity	Coverage breadth	Early‐training essentiality	Total score	Curriculum gap priority
Applied surgical anatomy	2	1	1	0	2	High
Applied surgical physiology	3	1	1	0	2	High
Applied surgical pathology	2	1	1	0	2	High
Microbiology	3	1	2	1	4	Moderate
Imaging and data/Audit	4	1	1	1	3	High
Common surgical conditions	6	1	1	0	2	High
Perioperative management	11	1	2	1	4	Moderate
Trauma	18	2	2	2	6	Low
Surgical care of children	3	1	0	0	1	Critical
Medico‐legal and patient safety	7	2	2	2	6	Low
Clinical and procedural skills	11	2	2	2	6	Low
Communication and professionalism	6	2	2	2	6	Low

### 3.2 Perioperative Nursing Curriculum Coverage

Mapping of SURGhub nursing courses against the IFNA curriculum showed a stronger representation of selected practical anesthesia‐related domains than of basic sciences, pharmacology, research, and organizational content. Basic principles of anesthesia and physiology had two mapped courses and were rated as high‐priority gaps, whereas pathophysiology had three mapped courses and was also rated as high‐priority. Several IFNA domains, including basic science, anesthesia pharmacology, advanced anesthesia pharmacology, research theory/masters project, economics and organization, and nurse anesthesia review, had no mapped courses and were rated as critical‐priority curriculum gaps. Advanced principles of anesthesia and pharmaco‐therapeutics had one mapped course, but both remained priority gaps because coverage was narrow or incomplete (Table 3).

**TABLE 3:** Level 1 and Level 2 perioperative nursing course mappings to IFNA curriculum domains.

Domains	Count of applicable courses	Relevant content quantity	Coverage breadth	Early‐training essentiality	Total score	Curriculum gap priority
CanMEDS roles, ethical, and legal aspects of nurse anesthesia	0	0	0	0	0	Critical
Basic principles of anesthesia	2	1	1	0	2	High
Basic science	0	0	0	0	0	Critical
Anesthesia pharmacology	0	0	0	0	0	Critical
Physiology	2	1	1	0	2	High
Pathophysiology I/II	3	1	1	1	3	High
Advanced principles of anesthesia I, II, and III	1	1	0	0	1	Critical
Advanced anesthesia pharmacology	0	0	0	0	0	Critical
Research theory/Masters thesis project	0	0	0	0	0	Critical
Pharmaco‐therapeutics	1	1	0	1	2	High
Economics and organization	0	0	0	0	0	Critical
Nurse anesthesia review	0	0	0	0	0	Critical

## 4 Anesthesia Curriculum Coverage

When mapped to the University of Papua New Guinea anesthesia training curriculum, SURGhub Level 1 and Level 2 anesthesia‐relevant courses showed good coverage of perioperative medicine, crisis management, infection control, and patient safety, but important omissions in core clinical domains. Preoperative assessment and perioperative medicine were the most densely covered domains, with 12 mapped courses, whereas safety and quality in anesthetic practice and resuscitation, trauma, and crisis management each had 10 mapped courses, largely driven by content on patient safety, error prevention, crisis resource management, and emergency care. Regional anesthesia and local anesthetics (7 courses), transfer medicine and trauma/stabilization (9 courses), intensive care medicine exposure (6 courses), and airway management (3 courses) were all represented and generally rated as moderate‐ to high‐priority, reflecting their central role in early anesthesia practice.

However, several fundamental domains had no mapped courses and were classified as critical curriculum gaps, including premedication, recovery and post‐anesthesia care, day surgery and ambulatory anesthesia, sedation and general anesthesia, and ophthalmology and anesthesia. Pain medicine was represented by a single *Essential Pain Management* course, and pediatric anesthesia was represented by two courses, *Blood Sparing Techniques in Pediatric Anesthesia* and *Perioperative Blood Management in the Pediatric Patient*. Both domains were classified as high‐priority gaps, reflecting the narrow coverage of these broad areas (Table 4).

**TABLE 4:** Level 1 and Level 2 anesthesia course mappings to PNG anesthesia training curriculum.

Domains	Count of applicable courses	Relevant content quantity	Coverage breadth	Early‐training essentiality	Total score	Curriculum gap priority
Premedication	0	0	0	1	1	Critical
Recovery and post‐anesthesia care	0	0	0	0	0	Critical
Emergency perioperative care (ASA 1E/2E)	2	1	1	0	2	High
Pediatric anesthesia (basics)	2	1	1	1	3	High
Day surgery and ambulatory anesthesia	0	0	0	0	0	Critical
Sedation and general anesthesia	0	0	0	0	0	Critical
Pain medicine	1	1	0	1	2	High
Professional skills, human factors, and wellbeing	3	1	2	1	4	Moderate
Obstetric anesthesia	4	2	1	0	3	High
Infection control	2	1	1	1	3	High
Intensive care medicine (basic exposure)	6	1	1	1	3	High
Induction, intraoperative care, and monitoring	3	2	1	1	4	Moderate
Resuscitation, trauma, and crisis management	10	1	1	1	3	High
Regional anesthesia and local anesthetics	7	2	1	1	4	Moderate
Airway management	3	1	1	0	2	High
Preoperative assessment and perioperative medicine	12	2	1	1	4	Moderate
Transfer medicine and trauma/Stabilization	9	2	1	1	4	Moderate
Safety and quality in anesthetic practice	10	2	2	1	5	Low
Ophthalmology and anesthesia (basic)	0	0	0	1	1	Critical

### 4.1 OBGYN Curriculum Coverage

Curriculum mapping to the RCPI Basic Specialist Training curriculum in OBGYN (Table 5) demonstrated limited coverage across most domains. Of 56 mapped competency domains, 37 were classified as critical gaps and 18 as high‐priority gaps, with only the caesarean section rated as a moderate gap and no domains rated as low‐priority gaps. Critical gaps were particularly concentrated in early pregnancy care, antenatal pathologies, obstetric emergencies, labor ward management, general gynecology, and contraception/family planning. Available content was concentrated in caesarean section, operative gynecology, infection control, wound care, communication, and management of acutely ill patients, but coverage remained narrow across most domains (Table 5).

**TABLE 5:** Level 1 and Level 2 OBGYN course mappings to RCPI BST curriculum.

Competency domains	Count of applicable courses	Relevant content quantity	Coverage breadth	Early‐training essentiality	Total score	Curriculum gap priority
General and clinical skills						
History taking	0	0	0	0	0	Critical
Physical and clinical examination	0	0	0	0	0	Critical
Wound care and management	2	1	0	1	2	High
Audit and quality improvement	0	0	0	2	2	High
Communication	1	1	1	1	3	High
Management of early pregnancy						
Early pregnancy problems	0	0	0	0	0	Critical
Care of patients with first trimester miscarriage	0	0	0	0	0	Critical
Care of patients with suspected or confirmed ectopic pregnancy	1	1	1	0	2	High
Antenatal care						
Common pregnancy problems	0	0	0	0	0	Critical
Antenatal care of a patient with previous caesarean section	0	0	0	0	0	Critical
Fetal assessment and CTG interpretation	0	0	0	0	0	Critical
Care of patient with diabetes in pregnancy	0	0	0	0	0	Critical
Care of patient with hypertensive disorders in pregnancy	0	0	0	0	0	Critical
Care of patient with small for gestational age fetus	0	0	0	0	0	Critical
Care of patient with obstetric cholestasis	0	0	0	0	0	Critical
Care of patients with antepartum hemorrhage	0	0	0	0	0	Critical
Care of patients with previous or suspected venous thromboembolism	0	0	0	0	0	Critical
Obstetric emergencies						
Care of patients with threatened or established preterm labor	0	0	0	0	0	Critical
Care of patients with cord prolapse	0	0	0	0	0	Critical
Management of shoulder dystocia	0	0	0	0	0	Critical
Care of patients with pre‐eclampsia and severe pre‐eclampsia	0	0	0	0	0	Critical
Management of maternal collapse	2	1	0	0	1	Critical
Management of postpartum hemorrhage	0	0	0	0	0	Critical
Labor ward and postpartum management						
Labor and delivery	0	0	0	0	0	Critical
Skilled birth attendance for obstetricians	0	0	0	0	0	Critical
Management of induction of labor	0	0	0	1	1	Critical
Caesarean section	4	2	2	0	4	Moderate
Dystocia in labor	0	0	0	0	0	Critical
Labor in the presence of a previous caesarean section	0	0	0	0	0	Critical
Operative vaginal delivery	0	0	0	0	0	Critical
Care of patients with perineal tears	0	0	0	0	0	Critical
Preoperative management	0	0	0	0	0	Critical
Care of patients with complications during the puerperium	0	0	0	0	0	Critical
General gynecology						
Preoperative care	0	0	0	0	0	Critical
Menstrual problems and abnormal bleeding, amenorrhea	0	0	0	0	0	Critical
Care of patients with acute pelvic pain and dysmenorrhea	0	0	0	0	0	Critical
Problems of the vulva and vagina	0	0	0	0	0	Critical
Menopausal problems	0	0	0	2	2	High
Care of patients with postmenopausal bleeding	0	0	0	0	0	Critical
Manage family planning/contraception	0	0	0	0	0	Critical
Gynecology subspecialties						
Operative gynecology	6	1	0	1	2	High
Urogynecology	0	0	0	2	2	High
Oncology	1	1	0	0	1	Critical
Infertility	0	0	0	2	2	High
Contraception/family planning	0	0	0	0	0	Critical
Core professional skills						
Good professional practice	0	0	0	0	0	Critical
Infection control	3	1	1	0	2	High
Self‐care and maintaining wellbeing	0	0	0	2	2	High
Communication in clinical and professional setting	3	2	0	1	3	High
Leadership	0	0	0	2	2	High
Quality improvement	0	0	0	2	2	High
Scholarship	0	0	0	2	2	High
Management	0	0	0	2	2	High
Standards of care	2	1	1	1	3	High
Dealing with and managing acutely ill patients in appropriate settings	3	1	1	0	2	High
Therapeutics and safe prescribing	2	1	1	1	3	High

## 5 Discussion

SURGhub is a major open‐access educational initiative for the global surgical team, particularly in low‐resource settings. Its rapid expansion demonstrates substantial global uptake and suggests that open‐access, low‐bandwidth digital platforms can address important barriers to accessing surgical and perioperative workforce education resources. This cross‐sectional curriculum‐mapping study established a baseline assessment of how SURGhub Level 1 and Level 2 courses aligned with selected early training curricula across surgery, anesthesia, OBGYN, and perioperative nursing. Curriculum coverage was uneven both within and between disciplines, but given that SURGhub was developed primarily as a curated, quality‐assured platform to improve access to existing educational material, rather than as a curriculum built de novo, some imbalance in content coverage is unsurprising.

Surgical content was concentrated in trauma, perioperative care, and patient safety with less representation of basic sciences, pediatrics, and common surgical conditions. Perioperative nursing content was better aligned to practical anesthesia‐related topics than to foundational sciences, pharmacology, and research. Anesthesia content similarly emphasized safety, crisis management, trauma, and perioperative assessment, with less coverage of routine core practice such as sedation, general anesthesia, post‐anesthesia recovery, and day‐case care.

Table 1 and Figure 1 summarize the curriculum coverage scores for each discipline. The higher surgical and anesthesia scores indicate closer alignment with their benchmark curricula than OBGYN and perioperative nursing. The low median scores in the latter disciplines indicate gaps across large sections of their respective training curricula supporting broader curriculum‐aligned commissioning alongside targeted expansion of critical and high‐priority surgical and anesthesia domains.

Despite the identification of three relevant surgical courses, the domain surgical care of children was classified as a critical‐priority curriculum gap because the courses provided a limited coverage of the broader competencies within the benchmark curriculum. Population‐based surveys from four LMICs found that 19% of children had a surgical need and that 62% of these needs were unmet [9]. Pediatric surgical care also requires age‐specific clinical and perioperative competencies that cannot be fully addressed through general adult surgical content. Population growth in LMICs has led to a substantial increase in the number of children living in these settings, making the unmet need for pediatric surgical and anesthetic care an important priority. Strengthening education and training for local healthcare providers has been identified as an important component of improving pediatric surgical capacity in LMICs [10]. Future SURGhub content development should therefore consider foundational pediatric surgical topics relevant to nonspecialist and early stage surgical trainees, focusing on initial assessment, perioperative care as well as recognition and management of common pediatric surgical conditions.

Compared with the other specialties, the available OBGYN courses covered a much smaller percentage of the chosen curriculum with many more high and critical priority gaps. SURGhub OBGYN content remains concentrated in procedural and perioperative domains, with limited coverage of antenatal pathology, early pregnancy care, obstetric emergencies, labor ward management, and general gynecology. This relative under‐representation is particularly important given the ongoing burden of maternal mortality and morbidity in LMICs where ninety‐four percent of maternal deaths occur with two‐thirds occurring in Sub‐Saharan Africa and one‐fifth in Southern Asia. Maternal mortality rates remain unacceptably high with greater than 800 maternal deaths occurring each day with hemorrhage, hypertensive disorders, sepsis, abortion complications, and thromboembolism among the leading direct causes [11]. These findings support prioritization of educational content for early stage OBGYN and nursing learners in future phases of SURGhub development.

O'Flynn et al. argue that technology can expand surgical training in low‐resource settings, but that success depends on findability, quality, contextual appropriateness, and sustainability. The authors also note that there is a surfeit of resources on some areas and a corresponding dearth of resources in others, meaning that learners may find it difficult to find high‐yield topics while at the same time encountering repetition of content which is unlikely to cover the subject area. Therefore, while the availability of online open access educational content is valuable, what is more valuable is content that is structured around learner needs [12].

These findings should be interpreted in the context of SURGhub's development as a curated, quality‐assured platform rather than a curriculum built de novo. Its rapid global adoption demonstrates the feasibility of delivering open‐access surgical education at scale, but the uneven distribution of content identified in this study shows that scale alone is insufficient. The next phase of platform development should therefore combine continued learner reach with deliberate, curriculum‐aligned content commissioning in domains identified as critical‐ or high‐priority gaps.

This study has several limitations. First, curriculum mapping was performed against selected benchmark curricula rather than a single universally accepted global framework, and different reference curricula may have produced somewhat different patterns of coverage. Second, although mapping was performed by reviewers with subject‐specific expertise, the assignment of curriculum‐gap priority was based on subjective judgment and may be influenced by differences in educational perspective. Third, the number of courses within a domain does not fully capture depth, quality, or pedagogical effectiveness of content. Fourth, this analysis assessed platform content availability rather than learner completion, knowledge acquisition, or impact on clinical practice. The rubric provided a transparent and structured approach to curriculum‐gap prioritization but has not been externally validated or formally tested for reliability. The rubric was developed specifically for this study and was applied without formal pilot testing or prior validation. Alternative weighting systems incorporating disease burden, mortality risk, procedural frequency, or regional training priorities may have produced different rankings. We used composite scores summarized using means and medians and alternative analytical approaches could treat the score as an ordinal measure, which may have produced different summaries of curriculum coverage.

Fifth, each discipline was primarily mapped and scored by one subject‐specific reviewer rather than by multiple independent reviewers. Consequently, differences in reviewers' clinical backgrounds, educational experience, and practice environments may have influenced course‐to‐domain assignments and rubric scores. Finally, all four reviewers had an existing relationship with SURGhub either through research involving the platform or as members of the project scope committee. This allowed for detailed contextual knowledge of the platform but may have introduced an expectation bias. Use of the predefined rubric and group discussion of unclear ratings were intended to improve consistency, but no independent external mapping was undertaken.

## 6 Conclusion

SURGhub has demonstrated the potential of open‐access, low‐bandwidth digital education to expand access to surgical learning at a global scale. This curriculum mapping study shows that despite broad multidisciplinary reach, Levels 1 and 2 content coverage remains uneven across benchmark curricula. Practical perioperative, trauma, procedural, and patient‐safety topics are better represented than foundational sciences and several specialty‐specific clinical domains, particularly in OBGYN and perioperative nursing. The next phase of SURGhub development should prioritize curriculum‐aligned expansion in domains identified as critical‐ or high‐priority gaps, while maintaining attention to learner reach, accessibility, engagement, and equity.

## Author Contributions

All authors participated in conceptualization, manuscript writing of the original draft, reviewing and editing, and have approved the final manuscript. **Dáire Goodman:** conceptualization, project administration, data curation, formal analysis writing – original draft, writing – review and editing, methodology, investigation. **Sara Mohan:** conceptualization, formal analysis, writing – original draft, writing – review and editing. **Pauline Wake:** conceptualization, formal analysis, writing – original draft, writing – review and editing. **Dolorence Wakida:** conceptualization, formal analysis, writing – original draft, writing – review and editing. **Dara Kavanagh:** conceptualization, review, supervision. **Juan Carlos Puyana:** conceptualization, writing – original draft, writing – review and editing, supervision. **Eric O'Flynn:** conceptualization, writing – original draft, writing – review and editing, supervision.

## Funding

The authors have nothing to report.

## Ethics Statement

This study used publicly available information on educational content and did not involve identifiable learner data, patient data, or intervention with human participants. Formal research ethics review and informed consent were therefore not required.

## Conflicts of Interest

All authors contribute to SURGhub in a professional or voluntary capacity. SURGhub is affiliated with the World Journal of Surgery. The authors declare no conflicts of interest.

## Supporting information


**Table S1:** Four reviewer profiles.

**Table S2:** SURGhub Level 1 and Level 2 courses included in the January 2026 curriculum‐mapping study.

## Data Availability

The data that support the findings of this study are available from the corresponding author upon reasonable request.
