# Supplementary material for: Aligning Open‐Access Surgical Education With Need: Curriculum Coverage and Gaps in the United Nations Global Surgery Learning Hub (SURGhub)
Source: World J Surg. 2026 Aug 13;50(10):2946–55. doi: 10.1002/wjs.70533 (PMC13662493; doi:10.1002/wjs.70533)
Supplement: Supplementary file 1 [file WJS-50-2946-s001.docx]

## Supplementary Table S1: Four reviewer profiles

| **Reviewer** | **Discipline mapped** | **Clinical specialty and seniority** | **Clinical and teaching experience** | **Country of practice** | **Relationship with SURGhub** |
| --- | --- | --- | --- | --- | --- |
| Dáire Goodman | Surgery | Specialist Registrar in Plastic Surgery | Medical school guest lecturer | Ireland | Doctoral candidate investigating the SURGhub platform |
| Sara Mohan | Obstetrics & Gynaecology | Research Fellow and Specialist Registrar in OBGYN | Medical student tutor | Ireland | Member of the SURGhub Project Scope Committee |
| Pauline Wake | Anaesthesia | Qualified Anaesthesiologist | Clinical lecturer | Papua New Guinea | Member of the SURGhub Project Scope Committee |
| Dolorence Wakida | Perioperative nursing | Head of Public Health Nurses College | Clinical lecturer | Uganda | Member of the SURGhub Project Scope Committee |
