# Supplementary material for: Aligning Open‐Access Surgical Education With Need: Curriculum Coverage and Gaps in the United Nations Global Surgery Learning Hub (SURGhub)
Source: World J Surg. 2026 Aug 13;50(10):2946–55. doi: 10.1002/wjs.70533 (PMC13662493; doi:10.1002/wjs.70533)
Supplement: Supplementary file 2 [file WJS-50-2946-s002.docx]

Supplementary Table S2. SURGhub Level 1 and Level 2 courses included in the January 2026 curriculum-mapping study. The table lists the unique eligible courses and the disciplines to which each course was mapped. A course could be mapped to more than one discipline. Discipline-specific course counts are therefore not mutually exclusive. Note: The table contains 82 unique Level 1 and Level 2 courses and 202 discipline-specific course assignments: surgery, *n* = 61; anaesthesia, *n* = 50; OBGYN, *n* = 65; and perioperative nursing, *n* = 26. The Perioperative Nursing e-Learning Foundational Programme contains 30 individual topics within one module

| No. | Course title | Surgery | Anaesthesia | OBGYN | Perioperative nursing |
| --- | --- | --- | --- | --- | --- |
| 1 | 1-Handed Knot-Tying | ✓ |  | ✓ |  |
| 2 | 2-Handed Knot Tying | ✓ |  | ✓ |  |
| 3 | Acute Burn Care | ✓ | ✓ | ✓ | ✓ |
| 4 | Adjuvant Medications for Peripheral Nerve Block |  | ✓ |  |  |
| 5 | Airway Matters |  | ✓ |  |  |
| 6 | All About Aseptic Technique | ✓ | ✓ | ✓ | ✓ |
| 7 | Anaesthesia for Free Flap Surgery |  | ✓ |  |  |
| 8 | Anaesthesia in War |  | ✓ |  |  |
| 9 | Animal Bite and Insect Sting | ✓ |  | ✓ |  |
| 10 | Appendicitis | ✓ |  | ✓ |  |
| 11 | Apprendre et Transmettre l’Anesthésie Locorégionale Échoguidée en Toute Sécurité – ADRALE / F2AR |  | ✓ |  |  |
| 12 | Approach to Caesarean Section | ✓ |  | ✓ |  |
| 13 | Basic Burn Management | ✓ |  | ✓ |  |
| 14 | Basic Principles of Wound Management |  |  | ✓ |  |
| 15 | Behind the Knife – Trauma Surgery Video Atlas – Femoral Vessels | ✓ |  | ✓ |  |
| 16 | Behind the Knife BIG T Trauma Series – ED Thoracotomy | ✓ |  | ✓ |  |
| 17 | Blood Sparing Techniques in Paediatric Anaesthesia |  | ✓ |  |  |
| 18 | Brain Death | ✓ | ✓ | ✓ |  |
| 19 | Brainstem Reflexes during Neurosurgery: An Overview | ✓ | ✓ | ✓ |  |
| 20 | Burns | ✓ |  | ✓ |  |
| 21 | Burns: Resuscitation and Anaesthetic Management |  | ✓ |  | ✓ |
| 22 | C-Arm Intraoperative Imaging for Ankle Fractures | ✓ |  | ✓ |  |
| 23 | Cardiac Trauma | ✓ |  | ✓ |  |
| 24 | Chemical and Radiation Injuries | ✓ | ✓ | ✓ |  |
| 25 | Cleft Lip and Palate | ✓ |  | ✓ |  |
| 26 | Communicating Effectively – American Society of Anaesthesiologists | ✓ | ✓ | ✓ |  |
| 27 | Communication Skills for Telephone Consultations | ✓ | ✓ | ✓ | ✓ |
| 28 | Concomitant Brain and Spine Trauma – Lessons from Public Health and Epidemiology Studies | ✓ |  | ✓ |  |
| 29 | CVAD Complications: Prevention and Management | ✓ | ✓ | ✓ | ✓ |
| 30 | Difficult Airway Algorithm 2023 – American Society of Anaesthesiologists |  | ✓ |  | ✓ |
| 31 | Distractions in the Operating Room | ✓ | ✓ | ✓ |  |
| 32 | Drug Errors in Anaesthesia 2023 |  | ✓ |  |  |
| 33 | Ectopic Pregnancy |  |  | ✓ |  |
| 34 | Emergency Checklists in Anaesthesia Practice | ✓ | ✓ | ✓ | ✓ |
| 35 | Epidemiology – Fundamentals of Patient Safety 2024 | ✓ | ✓ | ✓ | ✓ |
| 36 | Essential Burn Care | ✓ | ✓ | ✓ |  |
| 37 | Essential Emergency & Critical Care | ✓ | ✓ | ✓ | ✓ |
| 38 | Essential Pain Management | ✓ | ✓ | ✓ | ✓ |
| 39 | Facial Paralysis | ✓ |  | ✓ |  |
| 40 | Fracture-Related Infections – Prevention/Diagnosis/Treatment | ✓ |  | ✓ |  |
| 41 | Fundamentals of War Surgery | ✓ |  | ✓ | ✓ |
| 42 | Gallbladder Disease | ✓ |  | ✓ |  |
| 43 | Hand Washing, Asepsis, Disinfection and Sterilization |  | ✓ |  |  |
| 44 | Hemorrhage Control in the Prehospital Setting – Crash Savers Team | ✓ | ✓ | ✓ | ✓ |
| 45 | Implications of Anaesthesia on Cancer Surgery | ✓ | ✓ | ✓ |  |
| 46 | Infection Control and Prevention | ✓ | ✓ | ✓ | ✓ |
| 47 | Informed Consent and Team Communication during Caesarean Section | ✓ | ✓ | ✓ | ✓ |
| 48 | Introduction to C-Arm Imaging and Radiation Safety in the Operating Room | ✓ | ✓ | ✓ | ✓ |
| 49 | Introduction to Community Engagement and Involvement | ✓ | ✓ | ✓ | ✓ |
| 50 | Laparoscopic Appendectomy for Uncomplicated Appendicitis | ✓ |  | ✓ |  |
| 51 | Laparoscopic Pylomyotomy | ✓ |  | ✓ |  |
| 52 | Local Anaesthetic Systemic Toxicity | ✓ | ✓ | ✓ |  |
| 53 | Management of Accidental Intra-arterial Injections | ✓ | ✓ | ✓ | ✓ |
| 54 | Medication Error Prevention – Fundamentals of Patient Safety 2024 | ✓ | ✓ | ✓ | ✓ |
| 55 | Nasal Reconstruction | ✓ |  | ✓ |  |
| 56 | Navigating the Global Surgery Ecosystem | ✓ |  | ✓ |  |
| 57 | Nontechnical Skills and Crisis Resource Management in the Operating Theatre | ✓ | ✓ | ✓ |  |
| 58 | Obstetric Trauma | ✓ | ✓ | ✓ |  |
| 59 | Obstetrics in the Setting of Remote Retrieval Medicine |  |  | ✓ |  |
| 60 | Perioperative Blood Management in the Pediatric Patient |  | ✓ |  |  |
| 61 | Perioperative Management of Patients Undergoing Orthopaedic Oncological Surgeries | ✓ | ✓ | ✓ |  |
| 62 | Perioperative Medicine: An Overview |  | ✓ |  |  |
| 63 | Perioperative Nursing e-Learning Foundational Programme (PeN Programme) |  |  |  | ✓ |
| 64 | Postoperative Assessment |  |  |  | ✓ |
| 65 | Practical Considerations to Access the Epidural Space |  | ✓ |  |  |
| 66 | Preoperative Assessment | ✓ | ✓ | ✓ | ✓ |
| 67 | Preoperative Assessment of Functional Capacity | ✓ | ✓ | ✓ | ✓ |
| 68 | Rapid Sequence Induction | ✓ | ✓ | ✓ |  |
| 69 | Skin Cancer Reconstruction |  | ✓ |  |  |
| 70 | Surgical Instrument Testing and Inspection | ✓ |  | ✓ |  |
| 71 | Surgical Skills – Suturing – Vertical Mattress | ✓ |  | ✓ |  |
| 72 | Suture Technique | ✓ |  | ✓ |  |
| 73 | Suturing Horizontal Mattress | ✓ | ✓ | ✓ |  |
| 74 | Triage and Resuscitation | ✓ | ✓ | ✓ |  |
| 75 | Trocar Placement | ✓ |  | ✓ |  |
| 76 | Ultrasound Guided Femoral Nerve Block | ✓ | ✓ | ✓ | ✓ |
| 77 | Understanding Arterial Blood Pressure Measurement |  | ✓ |  |  |
| 78 | Understanding Capnography | ✓ | ✓ | ✓ | ✓ |
| 79 | Video Consultations | ✓ | ✓ | ✓ | ✓ |
| 80 | WHO Surgical Safety Checklist | ✓ | ✓ | ✓ | ✓ |
| 81 | Wound Healing after C-Section |  |  | ✓ |  |
| 82 | Z-Plasty | ✓ |  | ✓ |  |
